# Adult height of children born small for gestational age treated with growth hormone and gonadotropin-releasing hormone analogs in Southern Brazil

**DOI:** 10.20945/2359-4292-2023-0513

**Published:** 2025-02-11

**Authors:** Luís Eduardo Cruvinel Pinto, Adriane de Andre Cardoso-Demartini, Julienne Angela Ramires de Carvalho, Gabriela de Carvalho Kraemer, Rosana Marques Pereira, Aline Scheidemantel, Gabriel Junqueira Soares, Suzana Nesi-França

**Affiliations:** 1 Setor de Endocrinologia Pediátrica Professor Romolo Sandrini, Departamento de Pediatria do Complexo Hospital de Clínicas, Universidade Federal do Paraná, Curitiba, PR, Brasil

**Keywords:** Infant, small for gestational age, growth hormone, human growth hormone, adult, body height

## Abstract

**Objective:**

To evaluate adult height and identify the factors contributing to its achievement in patients born small for gestational age (SGA) treated with recombinant human growth hormone (rhGH).

**Subjects and methods:**

This retrospective study includes data of SGA children treated at a pediatric endocrinology center. Inclusion criteria were SGA birth (birth length and/or weight < -1.28 standard deviation score (SDS), absence of catch-up growth by the age of four years, rhGH treatment for more than 12 months, and recorded adult height. Birth size SDS was calculated using Intergrowth-21st (gestational age ≥ 33 weeks) or Fenton (<33 weeks) standards. Patients with uncontrolled chronic diseases, genetic syndromes, or growth hormone deficiency were excluded. An increase of 0.6 SDS or more was considered a positive response.

**Results:**

Twenty-four patients (14 boys) were included, with an average gestational age of 38.0 (range: 33.0-40.0) weeks, birth weight of -1.3 ± 0.9 SDS, and birth length of -2.4 ± 0.7 SDS. They were treated with rhGH starting at an average age of 10.3 ± 2.6 years for a duration of 5.4 ± 2.3 years. Height SDS increased from -2.6 ± 0.4 SDS to -1.2 ± 0.6 SDS, which was comparable to the target height SDS (-1.3 ± 0.9; p = 0.3). Although 18 children were classified as good responders, 6 did not achieve a final height SDS > -2.0. Adult height was correlated with the increment in height SDS and growth velocity during the first year of treatment. No significant differences were observed between children classified as SGA by birth weight or length < 10th percentile and those by weight or length < -2.0 SDS.

**Conclusion:**

In this cohort of children born SGA with short stature, rhGH treatment effectively improved adult height. Given the diverse causes of being born SGA, the response to rhGH therapy may vary.

## INTRODUCTION

Children born small for gestational age (SGA) exhibit higher mortality and perinatal complication rates (^1^). However, the definition of SGA has historically varied, with previous studies defining SGA based on whether a newborn’s weight or length was below the 10^th^, 5^th^, or 3^rd^ percentiles, or if they fell below a standard deviation score of -2 for their age and sex (^2^-^5^). The World Health Organization (WHO) initially defined SGA as a child born with a weight below the 10^th^ percentile (-1.28 standard deviation score, SDS) for their gestational age (GA), favoring this measure due to its ease of measurement compared to length (^6^). As an increasing volume of studies reported on the long-term outcomes of children born SGA, the first consensus from international pediatric endocrinology societies defined every child born with a length and/or weight below -2 SDS for sex and GA as SGA. This cutoff point efficiently identifies most children who require ongoing assessment of their growth (^2^,^7^).

Approximately 90% of SGA children experience spontaneous catch-up in weight and length, usually beginning around 12 weeks of age and concluding by 2 years of age (^8^). This catch-up growth has significant anthropometric and metabolic implications, with recent studies exploring factors influencing these outcomes, such as breastfeeding (^9^,^10^). Those who do not achieve adequate catch-up growth by 3 years of age face a sevenfold increased risk of short stature in adulthood (^8^). For children lacking appropriate catch-up growth, treatment with recombinant human growth hormone (rhGH) has been shown to improve growth velocity (GV) and adult height (AH), with an average increase of 0.6-1.8 SD (^8^,^11^,^12^).

While children born SGA are often grouped together, the condition is multifactorial (^8^). This heterogeneity accounts for the variable treatment responses observed among individuals and the diverse outcomes reported across studies (^11^). Several factors influence the response to rhGH treatment, including the child’s height SDS at the beginning of therapy, growth response during the first year of treatment, duration of treatment, higher length SDS at birth, and maternal height (^8^,^11^,^13^).

The Brazilian National Agency of Sanitary Surveillance (ANVISA) has approved recombinant human growth hormone for the treatment of children with short stature born SGA, as recommended by the Latin American Consensus on the care of SGA children (^14^). However, its nationwide implementation remains inconsistent, with availability varying across different states (^12^).

The primary objective of this study was to describe the adult height of SGA patients treated with rhGH at the Pediatric Endocrinology Unit of Complexo Hospital de Clínicas at *Universidade Federal do Paraná* (CHC-UFPR), a tertiary public health center in Paraná, Brazil. This data was compared to information available worldwide, as no studies to date have published on the height outcomes of SGA adults within the Brazilian population. The second objective was to identify variables associated with better outcomes from rhGH treatment in this patient population.

## SUBJECTS AND METHODS

### Study design and subjects

This study was a single-center, retrospective analysis that included children born SGA between 1993 and 2010, who had current or previous follow-up at a tertiary pediatric endocrinology referral center in southern Brazil. Patients were selected from the hospital database if they were registered with the International Code of Diseases, 10^th^ Revision (ICD-10) under P05.1.

The inclusion criteria were characterized by SGA birth, the absence of spontaneous catch-up growth (GV > 0 SDS for chronological age and sex by the age of 4) (^8^,^15^), persistent short stature (height < -2.0 SDS for age and sex), at least 10 visits to the pediatric endocrinology team, and rhGH treatment for a minimum of 12 months. SGA was defined as birth weight or length below -1.28 SDS (10^th^ percentile) for sex and GA (^6^,^11^). This criterion was adopted as many patients received treatment before the publication of the first SGA consensus by the Pediatric Endocrine Societies (^7^,^16^). Children were divided into group A, comprising patients with length and/or weight at birth below -2.0 SDS for sex and GA, and group B, consisting of patients with length or weight between -2.0 SDS and -1.28 SDS.

The exclusion criteria included the absence of AH information in medical records; incomplete or uncertain data on GA, birth length, or weight; uncontrolled chronic diseases that could affect growth; the use of medications that could influence growth, or the presence of a known syndromic cause for the growth impairment. All patients were initially screened for growth hormone deficiency, excluding those with a growth hormone (GH) peak < 5 ng/mL on any stimulation test (^17^). Furthermore, patients with precocious puberty, defined as puberty onset before 8 years for girls or 9 years for boys, were excluded from the analysis.

Birth anthropometry data were classified using the Newborn Cross-Sectional Study of the INTERGROWTH-21st Project for children born with GA ≥ 33 weeks (^18^).

The age at the onset of puberty was determined by reaching Tanner stage 2 (^19^,^20^). Adult height was defined as a GV of < 2 cm/year or a bone age of > 14 years for girls and > 16 for boys (^21^). Target height (TH) was calculated as follows: (maternal height + paternal height) / 2 ± 6.5 cm, according to sex (^22^).

Weight, length/height, and TH SDS were calculated using the WHO standards (^23^), and GV SDS was calculated based on the standards by Kelly and cols. (^24^), due to the absence of national standards. AH SDS was computed against the reference population in two ways: relative to the reference height of children at the same chronological age and relative to the reference height of adults (defined as 19 years for both boys and girls) (Table 1). AH is reported as the height SDS relative to adult standards to prevent downward skewing of SDS due to earlier completion of growth.

**Table 1:** Comprehensive overview of patient characteristics at baseline and across rhGH treatment

	All	Group A	Group B	P-value
Sex				
Male	14	12	2	-
Female	10	7	3	-
SGA cause				
Smoking	1	1	0	-
Preeclampsia	1	1	0	-
Multiple pregnancy	4	4	0	-
Unknown	18	13	5	-
Birth data				
Gestational age (wk)^*^	38.0 (33.0-40.0)	37.9 (33.0-39.6)	39.6 (37.6-40.0)	0.50
Birth weight SDS	-1.3 ± 0.9	-1.6 ± 0.71	-0.3 ± 0.9	0.001
Birth length SDS	-2.4 ± 0.7	-2.6 ± 0.7	-1.6 ± 0.3	0.002
Target height SDS	-1.3 ± 0.9^**^	-1.5 ± 0.5	-0.5 ± 1.2	0.04
Short parent (%)	13 (54.1%)	11 (57.8%)	2 (40%)	-
At treatment start				
Height SDS	-2.6 ± 0.4	-2.6 ± 0.4	-2.4 ± 0.4	0.27
Height velocity SDS	-0.9 ± 1.4	-1.0 ± 1.5	-0.5 ± 0.9	0.46
Age	10.3 ± 2.6	9.8 ± 2.6	12.2 ± 1.0	0.06
Follow-up				
Treatment duration (yr)	5.4 ± 2.3	5.9 ± 2.3	3.9 ± 1.2	0.07
Age at puberty onset (yr)	11.4 ± 0.9	11.4 ± 12.1	11.5 ± 0.5	0.80
Height SDS at puberty onset	-2.0 ± 0.8	-2.0 ± 0.8	-2.3 ± 0.4	0.41
rhGH dose in 1^st^ yr (mg/kg/day)	0.04 ± 0.003	0.04 ± 0.003	0.04 ± 0.003	0.35
Height velocity SDS in 1^st^ yr (cm/yr)^*^	1.6 (-0.1 to 6.6)	1.8 (-0.1 to 6.6)	1.3 (0.5 to 2.4)	0.50
Height SDS gain 1^st^ yr	0.6 ± 0.3	0.6 ± 0.4	0.5 ± 0.2	0.45
GnRH analog (n)	18 (75%)	13 (68.4%)	5 (100%)	-
GnRH duration (mo)	18.4 ± 7.9	19.0 ± 7.9	16.6 ± 8.6	-
Final data				
Age at last measurement	16.3 ± 1.1	16.3 ± 1.0	16.5 ± 1.3	0.70
AH SDS (for age)	-1.2 ± 0.6	-1.3 ± 0.7	-1.2 ± 0.3	0.73
AH (for adult)	-1.5 ± 0.7	-1.6 ± 0.7	-1.3 ± 0.1	0.47
Adequate response	18 (75%)	13 (68.4%)	5 (100%)	-

Note: Data are shown as absolute frequencies (%) or means (SD).

* median (min-max).

** n = 23.

AH: adult height; GnRH: gonadotropin-releasing hormone; rhGH: recombinant human growth hormone; SDS: standard deviation score; wk: week; yr: year; SGA: small for gestational age.

Bone age (BA) assessment was performed with a wrist X-ray and classified using the standards of Greulich and Pyle (^25^). BA was considered delayed when < -2.0 SD, advanced when > +2.0 SD, or adequate when between -2.0 SD and +2.0 SD for chronological age and sex. Insulin-like growth factor-I SDS was not obtained due to the use of multiple assays throughout the cohort’s duration (^25^).

Patients were treated with rhGH at the standard initial dose of 0.05 mg/kg/day, administered subcutaneously 7 days a week. Subsequent titration was based on clinical response, with a positive response to therapy defined as an AH SDS increase of at least 0.6 above their starting height SDS. This criterion was chosen because it corresponds to the smallest mean increment noted before this study (^26^). Gonadotropin-releasing hormone analogs (GnRHa) were added for those who began treatment after the onset of puberty and had an unfavorable predicted AH (<-2.0 SDS) at the time of puberty onset, based on the collaborative judgment of the pediatric endocrinology team.

### Statistical analysis

The Statsoft Statistica software (version 12.5) was employed for analysis. All data underwent normality assessment using the Shapiro-Wilk test. Baseline characteristics were presented as mean ± standard deviation (SD) for variables with a symmetrical distribution and as median (range) for variables with an asymmetrical distribution. Mean differences were evaluated using the Student’s t-test for paired or unpaired samples, as appropriate. The Mann-Whitney U test or Wilcoxon test was used for non-parametrically distributed variables, assessing mean differences of unpaired or paired samples, respectively. Pearson’s or Spearman’s correlations were used for parametric or nonparametric data, respectively. All statistical tests were conducted two-sided, with results considered statistically significant if *p* < 0.05.

### Patient consent and Information

The study received approval from the Hospital Medical Ethics Committee (CAAE no. 68241323.8.0000.0096). Written informed consent was obtained from the patients or their parents for subjects under 18 years of age.

## RESULTS

**Figure 1:**
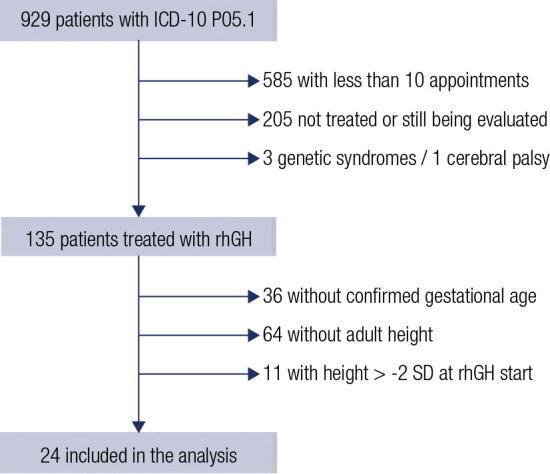
Flow chart of patient selection process.

ICD: International Classification of Diseases; SD: Standard Deviation; rhGH: recombinant human Growth Hormone.

Following the initial selection (Figure 1), 24 patients were included in the analysis, comprised of 14 males and 10 females. The baseline characteristics of these individuals are detailed in Table 1. Within this cohort, 5 patients were classified as SGA due to their birth weight or length being below -1.28 SDS (10^th^ percentile) (^6^,^11^). These patients received treatment before the publication of the first consensus and were included based on the WHO criteria available at that time (^7^,^16^). Notably, the majority of patients in the sample (79%) had birth weights and/or lengths below -2 SDS for their sex and GA.

The median gestational age was 38.0 (33.0-40.0) weeks, and 6 individuals were born prematurely (median GA of 35.5 weeks, ranging from 33.0 to 36.0 weeks). These patients exhibited similar responses to treatment as those born at term. The mean birth weight and length SDS were -1.3 ± 0.9 and -2.4 ± 0.7, respectively. Eleven patients were categorized as SGA for length, one for weight, and 12 for both. Parental height data were available for 23 patients, with the target height SDS being -1.3 ± 0.9. One patient had an unknown paternal origin (Table 1).

Treatment with rhGH commenced at an average age of 10.3 ± 2.6 years, starting with an initial dose of 0.05 mg/kg/day. The duration of treatment with rhGH averaged 5.4 ± 2.3 years, with a mean dose of 0.04 ± 0.003 mg/kg/day during the first year of treatment. The average onset of puberty was 11.6 ± 1.1 years in boys and 11.0 ± 0.5 years in girls. The height SDS at the onset of puberty was -2.0 ± 0.8. Eighteen patients (9 girls and 9 boys) received both rhGH and GnRHa therapy (Table 1). Girls experienced menarche at a median age of 14.2 years (range: 13.9-16.8 years), compared to their mothers, who experienced menarche at a median age of 12.5 years (range: 11.0-15.0 years). The AH SDS showed no significant difference between boys and girls (*p* > 0.05), with girls reaching an AH of -1.4 ± 0.6 SDS at an mean age of 15.5 ± 0.8 years, and boys achieving an AH of -1.6 ± 0.7 SDS at an mean age of 16.9 ± 0.8 years.

**Figure 2:**
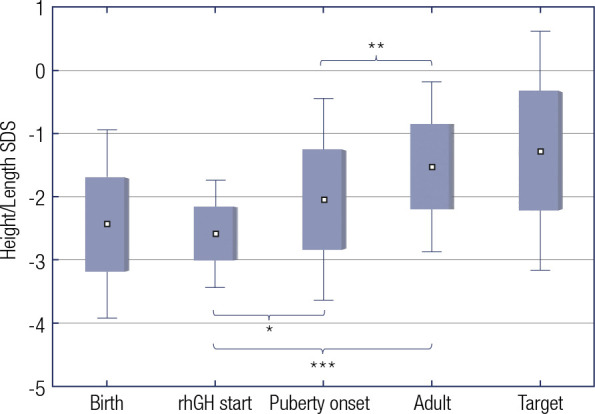
Height/Length SDS from birth to adult height. All results are presented as mean SDS.

No significant differences were observed between groups A and B, except for a higher TH in group B (Table 1). Consequently, outcomes were evaluated collectively. In this sample, patients experienced a significant increase in height-SDS from -2.6 ± 0.4 to -1.5 ± 0.7 (*p* < 0.001). Their height SDS at the onset of rhGH therapy was 1.4 ± 0.8 below their TH. Post-treatment, patients achieved a mean height comparable to their TH (SDS difference of 0.3; *p* = 0.3) (Figure 2).

*p = 0.001. **p = 0.01. ***p < 0.001 (student’s T test). rhGH; recombinant human Growth Hormone; SDS, standard deviation score.

Five patients (20.8%) exhibited delayed BA at the onset of treatment. These individuals had a lower GV SDS (-0.6 *vs.* -2.3; *p* = 0.01) prior to commencing rhGH therapy and demonstrated a significantly higher increase in height SDS (0.9 *vs.* 1.7; *p =* 0.01) compared to those without a BA delay. No significant disparities in birth length/weight, TH, height SDS before treatment, GH peak, age at puberty onset, duration of treatment, and adult height were found between those with and without a BA delay. None of the patients presented with advanced BA at the commencement of treatment.

**Figure 3:**
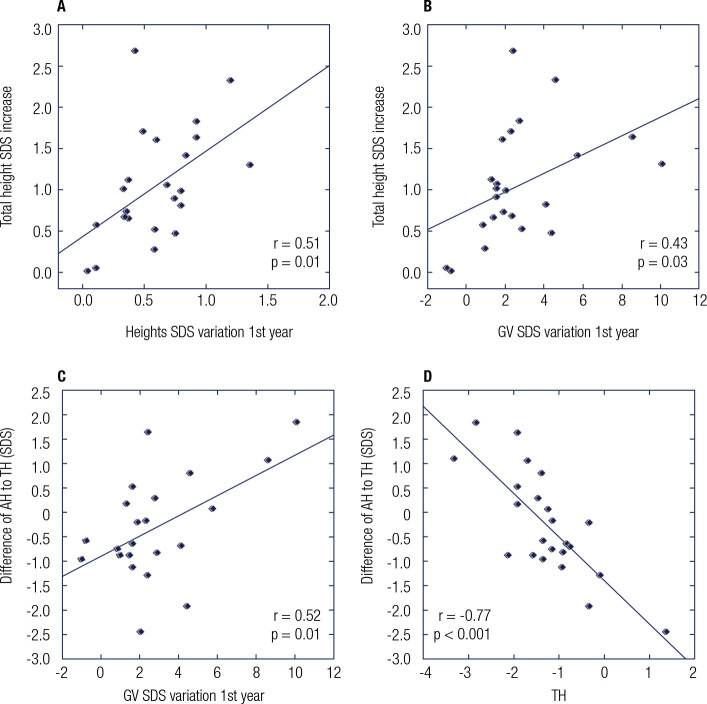
Variables associated to rhGH treatment response.

GV: growth velocity; AH: adult height; TH: target height; SDS: standard deviation score.

Correlations were explored between birth weight/length SDS, TH SDS, age at puberty onset, age at treatment start, and treatment duration with the increase in height SDS post-treatment; nevertheless, none of these variables were significantly associated with the height SDS increase (*p* > 0.05). An improvement in height SDS after the first year of treatment was significantly correlated with the overall increase in TH SDS. A negative correlation was observed between TH and adult height adjusted for parental height (r = -0.8; *p* < 0.001). Changes in growth velocity during the first year of treatment also correlated positively with the increase in height SDS and with adult height relative to TH (r = 0.5; *p* = 0.01) (Figure 3).

Eighteen patients (75%) were deemed good responders to rhGH therapy. Six patients did not achieve an adult height above -2 SDS, although three of them had good responses to the treatment. These patients presented with similar birth length/weight SDS, TH SDS, age at puberty onset, and duration of rhGH treatment as those who attained a normal adult height (*p* > 0.05). All three patients who exhibited a good response to therapy (with height SDS increases of 0.7, 0.9, and 1.1) but remained short post-treatment had severe short stature (height SDS < -3.0) prior to the initiation of rhGH therapy.

## DISCUSSION

This study, conducted within a subset of the Brazilian population, focused on children born SGA and followed up in a public health system hospital. It confirmed the effectiveness of rhGH treatment in promoting growth and improving adult height in children born SGA. Previous studies on AH after rhGH treatment in SGA children have reported an increase in height SDS ranging from 0.6 to 2.1 (^26^,^27^), with the majority indicating increases between 0.6 and 1.1 SDS (^11^,^15^,^28^-^31^).

The initiation of growth hormone treatment is recommended for SGA children who fail to exhibit catch-up growth by 2 years of age in Latin America and the USA (^7^,^8^,^14^,^16^), by 3 years in Japan, or by 4 years in Europe (^8^). However, in our study, the average age at the beginning of treatment was 10.3 ± 2.6 years, markedly higher than that recommended, a trend also observed in global reports, where the start age ranged from 6.6 ± 2.4 years to 12.7 ± 1.4 years (^26^,^27^). These findings, including those from our study, involve patients treated before the publication of the first consensus by the International Societies of Pediatric Endocrinology (^7^). Yet, more recent research indicates that initiating treatment between the ages of 2 and 4 years yields better outcomes (^32^,^33^).

The International Societies of Pediatric Endocrinology and the Growth Hormone Research Society advocate for beginning GH therapy between 2 to 4 years of age in SGA children with severe growth delays (height < -2.5 SDS) (^8^). The duration of treatment positively correlates with greater height SDS gains (^8^,^12^,^34^), and although the patients in this study underwent treatment for an average of 5.4 (±2.3) years, in line with previous studies (^11^,^15^,^26^-^31^), the duration of treatment did not significantly impact the variation in height SDS in our cohort.

Bone maturation has been deemed an unreliable predictor of pubertal timing or the attainment of adult height in children born SGA (^8^). However, a recent study found that SGA patients with more than two years of BA delay experienced a more significant increase in height SDS in the first year of treatment, including those without a GH deficiency (^35^). In our cohort, patients with delayed BA showed a higher total increase in height SDS, particularly in the first year of treatment. Previous suggestions indicate that SGA children with delayed BA do not undergo excessive progression in BA during the first year of rhGH treatment (^27^,^36^).

Interestingly, a longer prepubertal treatment period, which has been associated with better responses to rhGH, is crucial given that girls born SGA may experience earlier onset of puberty. Therefore, it is advised to monitor carefully during this life phase, and for children born SGA who remain short at the onset of puberty, with predicted AH < -2.5 SDS, the use of a GnRHa to postpone puberty for up to two years is recommended. In our study, 18 children received concurrent GnRHa therapy, likely reflecting the later age at treatment initiation.

Despite group B’s limited sample size, the improvements observed between the two groups were similar, suggesting that even though children born with weight and/or length < -2.0 SDS for sex and GA face a higher risk of adult short stature, those born smaller than < -1.28 SDS (10^th^ percentile) and with persistent short stature after 4 years of age may still benefit from rhGH treatment. This might be attributed to the diverse etiologies and the arbitrary nature of SGA classification based on the chosen birth size reference chart. Thus, it is plausible to conclude that variations in the causes of SGA birth could lead to differences in later-life outcomes and responses to treatment.

In this study, a significant correlation was observed between the increase in height SDS during the first year of treatment and the TH SDS gains, as highlighted in previous studies (^8^,^32^,^33^). However, this cohort’s data did not confirm a correlation between birth weight/length SDS, TH SDS, age at puberty onset, or treatment initiation, and the extent of increase in the patients’ height SDS.

Maternal height, signified by TH or maternal height itself, has previously been linked to greater height SDS gains during rhGH therapy (^8^). Contrary to these findings, the current study did not establish direct links between TH and AH or treatment outcomes. Instead, an inverse correlation was observed: the shorter the parents, the more pronounced was the exceedance of AH over TH or the lesser the height deficit of the patient relative to their genetic potential. This discrepancy may be due to untreated conditions among many parents that could interfere with TH. Moreover, the improvement in sanitary and health conditions in Brazil over recent decades, among other factors, might have contributed to an increment in population height, a phenomenon recognized globally as the secular growth trend (^37^).

The dosage of rhGH is a critical aspect potentially influencing growth responses; however, its evaluation in correlation with growth outcomes was impractical in this study due to the minimal variance in the sample’s average dosage. It is noteworthy that no substantial differences were discernible between patients characterized as good responders and non-responders. Recent advancements have clarified many previously unidentified genetic conditions that lead to SGA births and short stature. Nevertheless, the access to genetic testing, such as next generation sequencing panels or whole exome sequencing, has not become routine in Brazilian public healthcare.

This study faced several limitations, including the small sample size after the application of exclusion criteria and its retrospective nature. The absence of a control group of adult patients born SGA who did not undergo rhGH treatment posed another limitation. Most subjects were born SGA for unknown reasons, suggesting potential undiagnosed genetic alterations, which could be explored in future research. Notably, this cohort is distinct from previous ones in that it included patients with birth length and/or weight from -2.0 to -1.28 SDS. Adler and cols. (^11^) reported on a French cohort with similar inclusion criteria but did not exclude patients with conditions leading to short stature, thus possibly including those with chromosomal abnormalities or GH deficiency.

A considerable number of patients in this study were treated with GnRHa in conjunction with rhGH therapy, attributed to the later start of treatment and the fact that many (62.5%) had not achieved normal height by puberty onset. Further investigation is needed to elucidate the benefits of this therapeutic combination; however, its inclusion is advocated by the current international consensus (^8^) for patients commencing puberty with a height SDS < -2.5 or initiating treatment post-puberty onset (^38^). In this cohort, GnRHa was considered for patients with a height-SDS < -2.0 at puberty onset or for those whose treatment started after puberty, based on clinical discretion due to the lack of explicit guidelines at the time. This approach might also explain the above-average age of menarche among girls.

In conclusion, patients born SGA exhibited improvement in AH SDS following rhGH therapy, with more pronounced benefits in those showing greater height or growth velocity increase in the first treatment year. This study is the first Brazilian research to report on the efficacy of rhGH therapy and the achieved AH in patients born SGA. Despite the limited sample, the outcomes were consistent among children defined as SGA either by a birth weight or length below the 10^th^ percentile or by weight and/or length below -2.0 SDS. This suggests that children born with weight or length between -1.28 and -2.0 SDS may also warrant consideration if they do not adequately catch up and exhibit short stature. It is important to note that rhGH treatment is not widely accessible across the country currently, and the initiation of treatment was not ideal, largely owing to delayed referrals to the pediatric endocrinology center.
